# The stabilization and destabilization of marine carbon observations: Co-producing knowledge in murky waters

**DOI:** 10.1007/s40152-025-00435-y

**Published:** 2025-07-15

**Authors:** Ramona Haegele

**Affiliations:** 1 Faculty of Arts, University of Bonn, Bonn, Germany; 2 German Institute of Development and Sustainability (IDOS), Environmental Governance and Transformation to Sustainability, Bonn, Germany

**Keywords:** Agential realism, STS, Marine carbon, Geopolitics, Ethnography

## Abstract

This article examines the complexities of marine carbon observations by exploring how non-humans and humans, including: scientists, floats, and geopolitics, (de-)stabilize these processes. Drawing on ethnographic fieldwork in Brazil and Germany, the study uses Karen Barad’s (2007) concept of agential realism to understand how these diverse actors are mutually co-producing knowledge on marine carbon. Instead of viewing entities as separate, intra-action emphasizes their co-constitution. Through theme-based coding, the analysis identifies both stabilizing and destabilizing forces in marine carbon observations. Stabilizing forces include the dedication of scientists, two-way communication between floats and humans, and the global accessibility of data on marine carbon observations. In contrast, destabilizing forces involve climate change’s impact on data collection and quality, funding shortages, and national borders. The research highlights how geopolitical and scientific practices are deeply dynamic and often overlooked in discussions of marine carbon observations. By following non-humans and humans and incorporating diverse perspectives from the sea and land, the study provides new insights into the (un-)becoming of marine carbon observations, emphasizing the importance of the more-than-human in shaping knowledge production practices. This work underscores the value of thinking with Science and Technology Studies and new materialism about marine environments.

## Autonomous diving buoys: An introduction to marine carbon observations

**Fig. 1:**
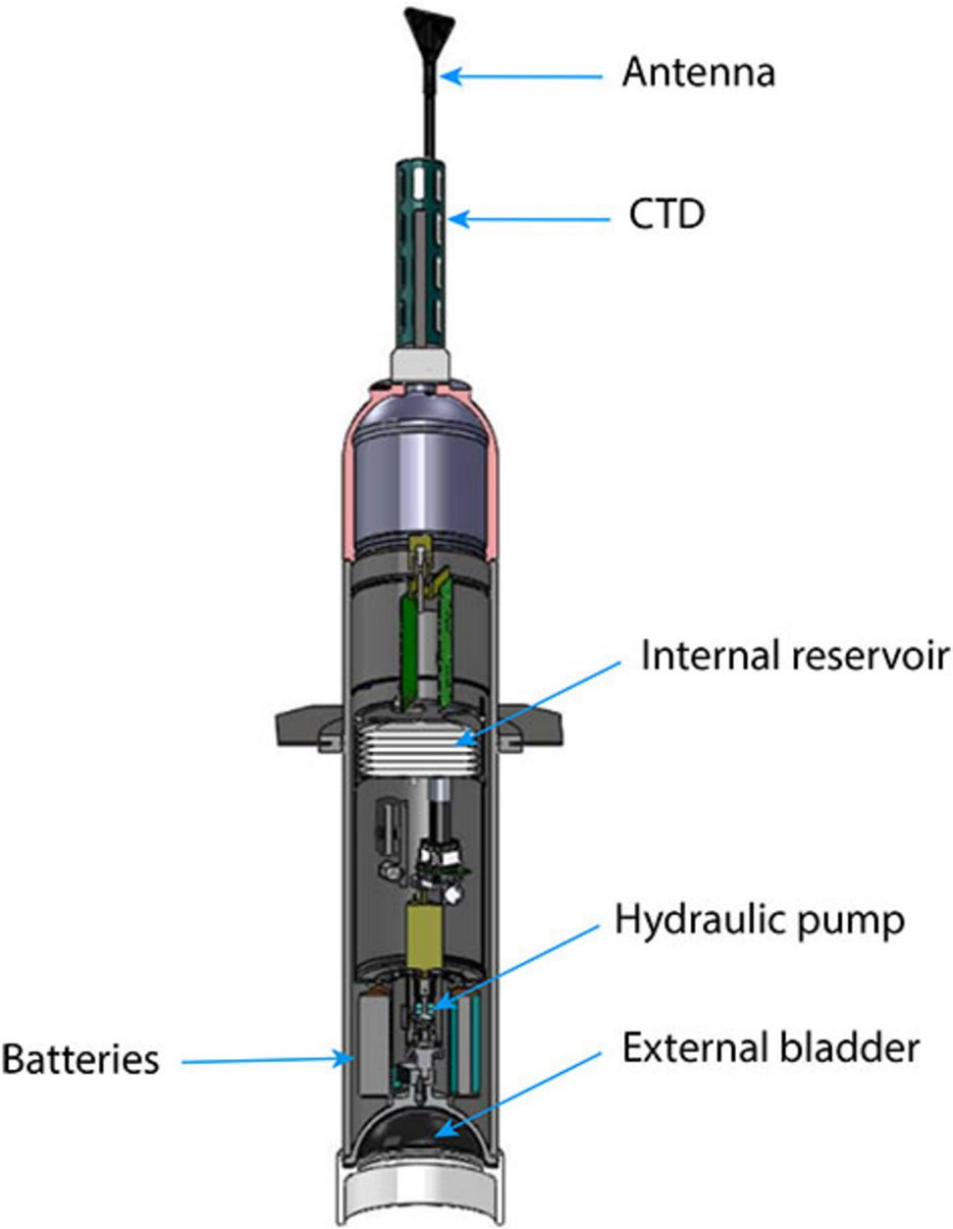
Argo float schematic (Argo 2023)

Imagine four thousand Argo floats^1^ moving up and down the ocean while you are reading this. Standard Argo floats measure hydrographic properties in the water, especially temperature and salinity at different water depths and thus provide observations on climate change in the areas of global warming, sea levels, and water cycle. These floats are autonomous diving buoys (Fig. 1) and are a more recent means in measuring marine carbon, by slowly adding biogeochemical sensors thereby providing data on ocean acidification and carbon fluxes.^2^ Marine carbon observations (MCOs) are crucial to understanding the ocean’s health, and its current, past, and future condition. These global observations feed into calculations for climate change scenarios and provide answers to a future ocean in a changing climate.

When I,^3^ as a social scientist, started to research knowledge production processes in MCOs within an interdisciplinary Brazilian and German joint research project in early 2021, I thought that marine carbon would be easy to determine. I imagined that a simple sensor for its measurement would exist. During research, however, I learned that it was not that easy, as there is no existing sensor which simply provides data on carbon levels in the ocean. Scientists, like oceanographers or biogeochemists, who are interested in determining marine carbon, must proceed by measuring four different parameters in a specific layer and space of the ocean, namely dissolved inorganic carbon, alkalinity, partial pressure of carbon dioxide, and pH-level. If one can determine two of these four parameters, the other two can be calculated afterwards (Byrne et al. 2010; Dickson et al. 2007; Steinhoff et al. 2019).

Argo floats are usually deployed in the open ocean from a vessel (Picture 1). After deployment, they move up and down different water columns measuring the temperature and salinity of the upper 2,000 m. To extend temperature and salinity measurements, Biogeochemical (BGC-) Argo floats have been developed. They measure, depending on the sensors they are equipped with, six additional properties, i.e. nitrate, oxygen, chlorophyll fluorescence, optical backscattering, downwelling light, and pH, and they can dive to a depth of 2,000 m (Bittig et al. 2019; Bundesamt fuer Seeschifffahrt und Hydrographie (BSH) 2024). While MCOs were predominantly conducted from ships in the past, advances in the miniaturization of sensors, significant improvements in data storage, and highly durable batteries have enabled remote and autonomous sensing (Lehman 2018). Currently, approximately four thousands floats are actively involved in measuring marine carbon, sending their data usually after a ten day drift via satellite to respective data centers (Bundesamt fuer Seeschifffahrt und Hydrographie (BSH) 2024).

**Picture 1:**
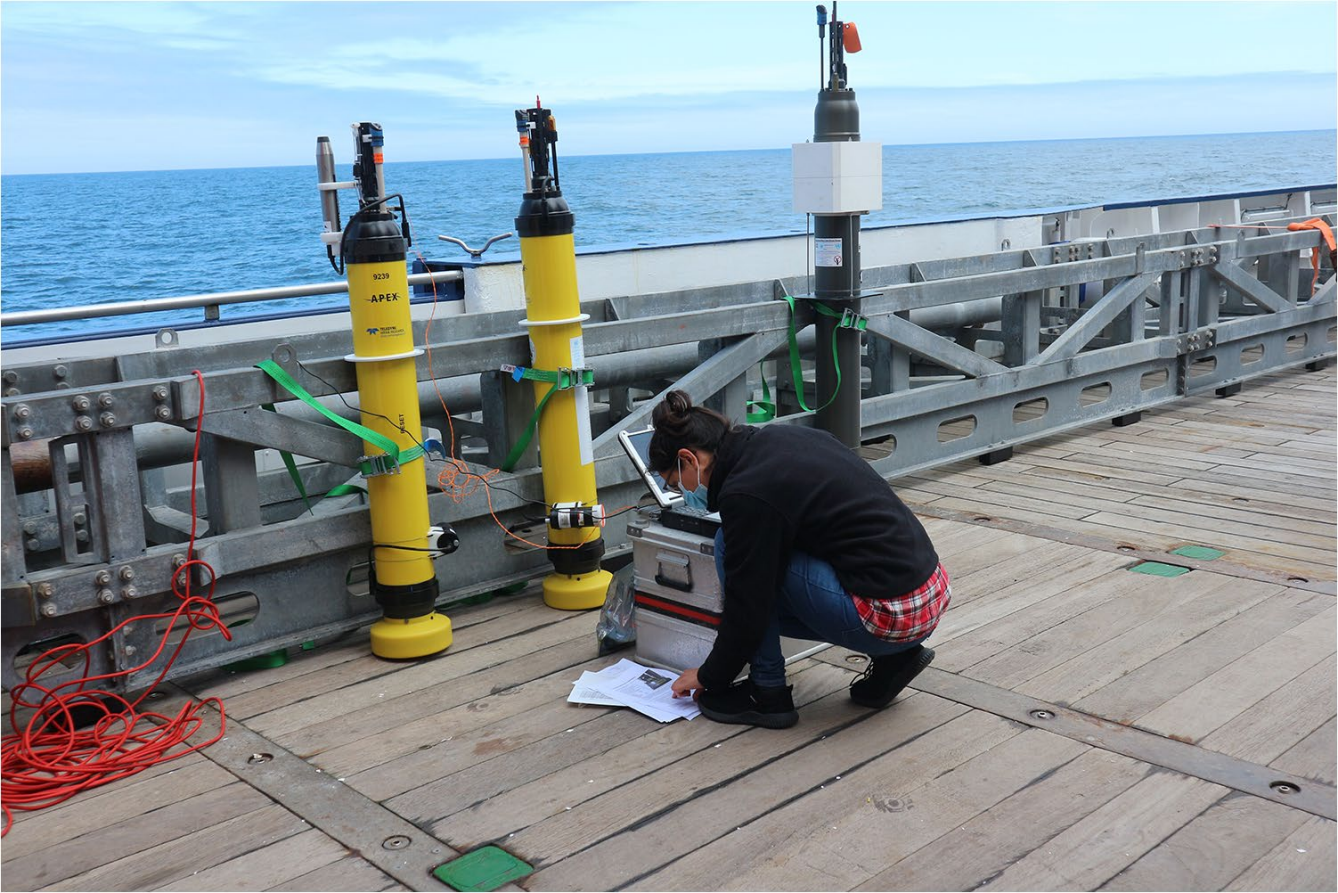
Author prepares the deployment of three (BGC-) Argo floats on a research vessel (Credit: Krastel 2021)

Scientists use this data to calculate and predict essential information for climate change scenarios, including those outlined in reports by the Intergovernmental Panel on Climate Change (IPCC) and the Global Carbon Budget (Friedlingstein et al. 2023; Pörtner et al. 2019). These measurements play a vital role in global climate negotiations and policymaking (Haegele and Schoderer 2021). Despite their importance and complexity, MCOs are still considered a niche topic in marine natural sciences (Aricò et al. 2021; Riebesell et al. 2009; Steinhoff et al. 2019). This is even more so in marine social sciences, where economic, geographical, or political science perspectives, for example, to assess the effectiveness of carbon dioxide removal, its social considerations and best practices (Boettcher et al. 2023; Satterfield et al. 2023; Sovacool et al. 2023), are the dominant topics in research.

Having spent over a year in the field between 2021 and 2023, including time on a research vessel, two merchant vessels, in laboratories, in marine science departments, and at conferences all focusing on MCOs, I am intrigued by the knowledge production processes within MCOs. As a social scientist interested in these practices within knowledge production, the following dynamic forces caught my attention: non-humans and humans. Non-humans include climate change, technologies, floats, satellites for data transmission, and software for data translation, funding of associated technologies and the deployment of floats in exclusive economic zones (EEZs), and humans, such as the scientists and technicians who can (not) measure marine carbon. I often wonder: How do scientists responsible for determining marine carbon navigate the methodological and technological challenges and uncertainties they encounter? How do they collect, interpret, and share data, or, in other words, how is knowledge about marine carbon (socially) produced? And how are the forces of non-humans, humans, and geo-politics implicated in (de-)stabilizing MCOs? In short, and this is the main question this article seeks to answer, how are MCOs (de-)stabilized?

So far, little is known about what (de-)stabilizes the knowledge production processes within MCOs. To address this gap in research, I propose approaching this topic by understanding non-humans and humans as intra-acting forces (Barad 2007) in stabilizing and destabilizing them. By examining the practices within MCOs through ethnographic fieldwork and by using the example of Argo floats, I explore non-human and human forces at play. I render Barad’s concept of intra-actions valuable for this article and, thus, my research contributes to approaches of marine Science and Technology Studies (STS) and oceanic new materialism (Fox and Alldred 2017; Harris 2021; Jensen 2017; Latour 1987).

MCOs exemplify key characteristics of current climate science: they function through automation, operate at a distance, are distributed across various sites, and are embedded in political contexts. While they play a growing role in observing the ocean, an analysis of their socio-material embeddedness remains widely unexplored. This paper approaches MCOs not as passive data-gathering tools, but as components actively involved in shaping knowledge and practice. Building on Karen Barad’s agential realism, I suggest that MCOs participate in producing specific understandings of marine environments, which have tangible effects, such as legitimizing conservation strategies, guiding financial allocations, or underpinning territorial claims. MCOs emerge at critical junctions where marine monitoring, ocean governance, and climate futures intersect. A Barad-inspired social science lens highlights how oceanic knowledge and control are co-constituted through entanglements of human and non- human forces.

The analysis builds on ethnographic fieldwork, including semi-structured interviews, participant observation (Bernard 2002), and go-alongs (Kusenbach 2003). Ethnography enabled me to follow (Marcus 1995) scientists in their daily work routines, following floats and humans to their diverse (geographical) stations on land and at sea, keeping abreast of the geopolitics involved in these processes, and helping to include multiple sites of meaning-making (cf. Haegele 2024; Mielke and Hornidge 2017; McAdam-Otto and Nimführ 2021).

To answer the main research question of how MCOs are (de-)stabilized, the article is structured as follows: The subsequent literature review discusses the STS and new materialism approaches which inspired my analysis. I present the conceptual framework, which proposes that MCOs can be perceived as intra-actions informed by Barad’s concept of agential realism (1998, 2007). The following section discusses the methods used for data collection and analysis of the empirical data. The next section presents the findings of MCOs as intra-actions and their (de-)stabilization through non-human and human forces. The conclusion demonstrates the dynamism of these forces and highlights the complexity of knowledge production processes within MCOs, which demonstrate that clear boundaries cannot be drawn to separate these forces.

## Conceptualizing marine carbon observations through a Baradian lens

More recently, the world’s ocean has become increasingly relevant as an object of study in the social sciences, often critically reconceptualizing the universal understanding of the ocean as static and usually associated with land, justice issues, and its *more-than-wet* materiality (Anderson and Peters 2016; Hein et al. 2024; Peters and Steinberg 2019; Tafon et al. 2023). Instead, more nuanced understandings of the ocean and the knowledge produced on the latter have been discussed, such as predominantly ship-based historiographic studies by Sorrenson (1996) or Laloë (2012), sociological research focusing on marine knowledge production processes by Hornidge (2020, 2018) or Bogusz (2018), or social network analysis on vessels pointing to the hierarchies at sea (Bernard and Killworth 1973).

Ocean observations have recently become an area of interest within the research on oceans in the social sciences. One research strand focuses on the global governance of oceans, with, for example Lehman (2016) referring to the Global Ocean Observation System (GOOS) to demonstrate how ocean observations create a digital twin of the sea, by conceptualizing the ocean as a space of potential for geopolitics and capitalism. Vadrot and Wanneau (2024) use an international relations perspective to analyze the politics of marine biodiversity, keeping track of the politics and practices of monitoring marine life, shedding light on inequalities in governing the ocean. Miloslavich et al. (2019) point to the need of expanding human capacity for ocean observations beyond scientists from financially well-resourced countries, including the global community through funding models, partnerships, and marine technology transfer.

A second research trend focuses on how scientists generate and manage data from ocean observations. Lehman (2018), sheds light on the challenges and opportunities of shifting from ship-based oceanography to partly autonomous remote data collection. This shift not only provides opportunities for more equitable scientific practices, such as access to data, but also comes with the loss of time at sea for scientists and widespread nostalgia about a bygone era of ship-based observations among oceanographers. Similarly, Helmreich (2009, 149) refers to scientists having access to data on the deep sea through robotic vehicles while being physically at home as “intimate sensing”. By following scientists’ knowledge production processes in mapping a deep-sea channel and its important role in storing carbon, Haegele (2024) identifies two modes of knowing the ocean – sensory landscapes, and experiential knowledge, as scientists rely on their own senses and experiences in science-making.

A third trend, which is also propounded by Helmreich (2023), addresses the agencies of non-human actors in the field of marine social sciences. Gramaglia and Mélard (2019) include more-than-humans, such as eels as a sensor for monitoring marine pollution to detect environmental change and toxicity. Jue (2014) considers the different scales of seawater through the agency of *Google Ocean* and *ATLAS *in silico, two data visualizations on ocean observations. In another study, Helmreich (2019) analyzes a wave buoy as part of ocean observations, its ability to monitor waves and its contribution to inequality between state organizations, militaries, multinational corporations, and citizens. Apostle and Gazit (2016) point to the dominance of political economies in tracking and observing marine species at risk.

This article builds and expands on these works. Barad’s framework of agential realism offers a crucial extension to these debates by emphasizing the relational and discursive dimensions of knowledge production. It shifts the focus from representation to the socio-material practices through which MCOs bring certain ocean and climate futures into being. This perspective enables an analysis of the (de-)stabilizations involved in making marine carbon comprehensible. By attending to the entanglements of non-human and human forces, a Baradian analysis highlights the dynamic processes through which MCOs contribute to shaping the ocean’s role in climate governance. In so doing, the article also expands on existing and highly relevant literature on the governance of ocean observations within the social sciences (e.g. Lehman 2016, 2018) by suggesting conceptualizing MCOs as intra-actions, hence strengthening new materialism approaches in the research field (Brennan 2018). The aim of approaches within this research agenda is to consider non-human and human forces in science-making through a relational approach. The article offers ethnographic depth of socio-material entanglements in the knowledge production processes on MCOs. The perspective I rendered valuable for the analysis is rooted within the framework of agential realism, which serves as a lens to include the indivisibility between the ethical and (geo-)political in which open-ended forces act dynamically. Barad (2007) does not separate epistemology from ontology or the human from the non-human, and in this understanding, materiality is not separate from, but entangled with the human world. Barad (2007) and other scholars from within new materialism, such as Braidotti (2013), describe a continuous nature of matter and meaning. Barad introduces the idea of the “dynamism of forces” (Barad 2007, 141), to argue that reality is not composed of separate, individual entities that simply interact, but Barad views the becoming of reality as a dynamic process (Barad 2007; cf. Law 2008).

Barad’s agential realism consists of: 1) apparatuses, 2) intra-actions, 3) phenomena, 4) agential cuts, and 5) diffractions. First, apparatuses are not mere instruments, but they “iteratively reconfigure space–time-matter as part of the ongoing dynamism of becoming” (Barad 2007, 141). Barad emphasizes that matter is already entangled with discourse in the enactment of phenomena. Thus, the apparatus of marine knowledge production is material-discursive, producing phenomena in an open-ended process through intra-actions of diverse forces, e.g. scientists and technologies.

Second, within intra-actions, non-humans and humans gain agency. In this line of thought, agency is emergent and not something an individual or an object possesses (Barad 2007), which differentiates this approach from a human-centric and linear thinking about agency (see Table 1). I found thinking about MCOs as intra-actions (Barad 2007) helpful, as I discovered that they are enacted by entangled non-human (predominantly floats) and human (mostly scientists and technicians) forces.

**Table 1:** Concepts of interactions and intra-actions (own elaboration based on Barad (2007, 2012), Bozalek and Fullagar (2021) and McAdam-Otto (2023))

Concept of	Interactions	Intra-actions
Actors	Preexisting and pre-established bodies or entities	Agency is not an inherent, determinate property of an actor but a “dynamism of forces”
Action	Entities meet, exchange and interact	Non-humans/humans do not gain agency; agency emerges in intra-actions of non-humans and humans
Process	Humans and non-humans interact, thereby modifying and changing with each other	Non-humans/humans permanently co-constitute and coproduce each other in an open-ended process

Third, a phenomenon produced by the apparatus “is a specific intra-action of an ‘object’; and the ‘measuring agencies’; the object and the measuring agencies emerge from, rather than precede, the intra-action that produces them” (Barad 2007, 128). MCOs thus constantly emerge through the dynamic forces of non-humans and humans at play which simultaneously produce these. This constant process of *becoming* is accompanied with the fusion of ontology, epistemology, and axiology – Barad’s ethico-onto-epistem-ological approach, highlighting the co-becoming of phenomena at play and the inseparability of intra-acting forces.

Fourth, agential cuts within the apparatuses draw boundaries between the subject (observers) and object (observed). My role as a researcher becomes central as I practice boundary-making in my doings, and in so doing diffract different types of agencies. Fifth, diffraction also serves as a methodology to cross the boundaries between disciplines aiming to rethink the old anew, potentially achieving unexpected outcomes, and becoming aware of diverse knowledge making processes (Barad 2007; Barla 2023).

With the argument that knowledge is not merely a reflection of a pre-existing world but part of the world’s becoming, I am well aware that I, the author of the article, am part of the apparatus and so is my methodology, the objects I observe, and the social and political situatedness of the research. I also contribute to the enactment of MCOs, as I am part of the becoming of the world (cf. Callon 1984; Fox and Alldred 2017; Haraway 1988). Thus, I want to note that my doings as a social scientist can lead to agential cuts as an act of observation of what is included and excluded (cf. Barad 2007). To be able to analyze the forces at play in enacting MCOs, I rely on collected qualitative data by using a multi-sited ethnographic approach, as outlined in the following chapter.

## Diving deep: An ethnographic approach towards marine carbon research

MCOs play a crucial role in our understanding of ocean acidification and climate change, yet they present unique challenges in implementation and interpretation. Scientists grapple with the inherent complexities of measuring carbon in dynamic marine environments, balancing the need for data availability with the limitations of current technologies and funding. Both the collection of data about marine carbon and its interpretation involves navigating uncertainties and variabilities across different temporal and spatial scales. Ethnographic research in this field can illuminate how non-humans and humans (de-)stabilize MCOs. Ethnographic case studies can reveal the socio-technical entanglements within the apparatus of marine knowledge production that enact MCOs and how they shape our understanding of oceanic processes and their role in the global carbon cycle.

Between April 2021 and March 2023, I conducted ethnographic fieldwork in Germany, Brazil, and in the Labrador Sea, the North Atlantic, the Baltic Sea, and along the Brazilian Coast^4^ for a period of one year. As common in STS research, it quickly became apparent that conducting research on knowledge production processes on MCOs has its own challenges, such as power-laden negotiations within research teams, epistemic authority, and depending on the research locality, “working and living outside of everyone’s comfort zone” (Hornidge 2018, 436). Hornidge’s (2018, 2020) foundational work and the idea of combining interviews with natural scientists, photo and video documentation of everyday routines in the field, and participant observation during work processes served as an inspiration for data collection on the production of scientific knowledge.

**Fig. 2:**
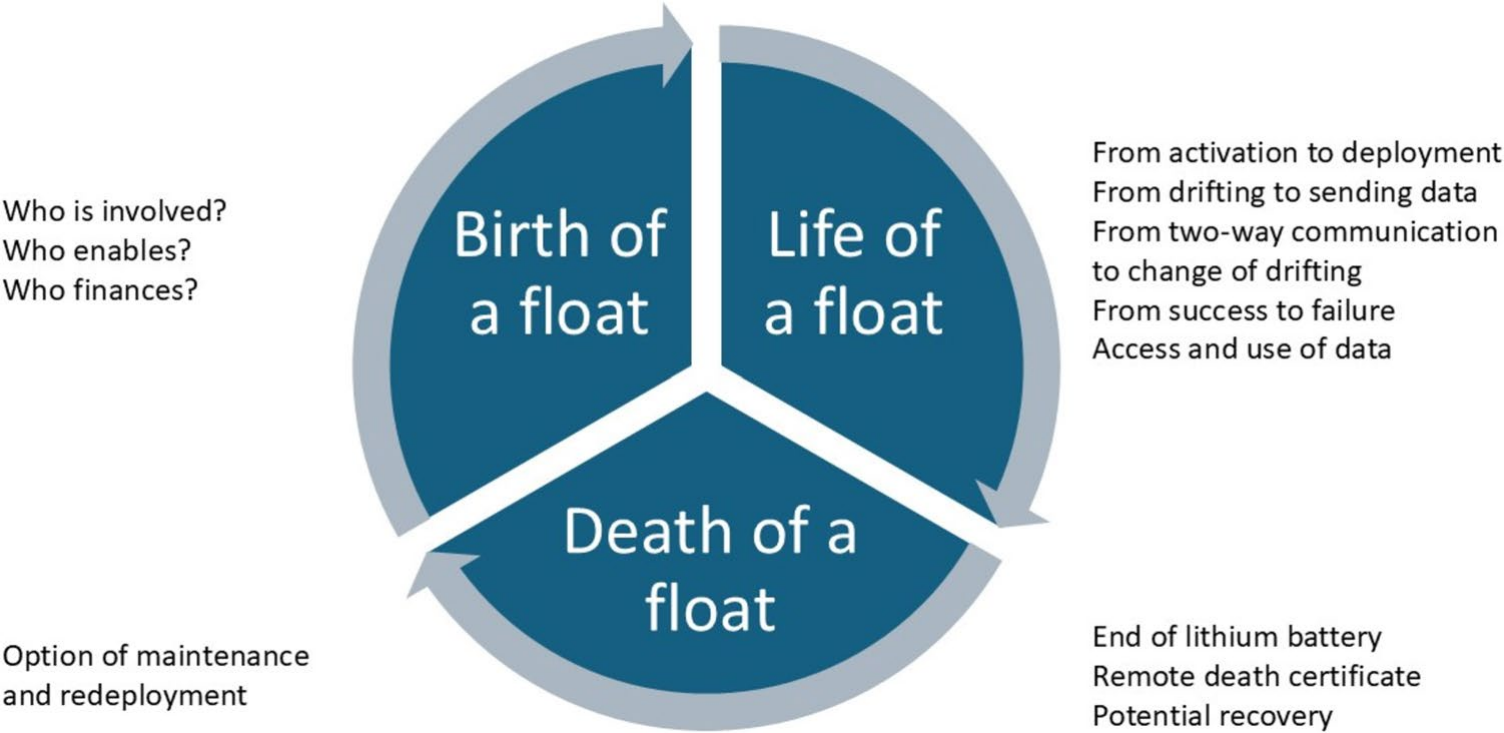
Life cycle of a float (own elaboration)

Informed by Marcus (1995) multi-sited ethnography approach, I followed (BGC-) Argo floats while they were brought to life (birth) in German marine science institutes^5^; I followed them during their lifetime from their deployment on a ship to their data delivery via online tools, and sometimes until their death when they were no longer sending any data via GPS (Fig. 2). Go-alongs (Kusenbach 2003) and participant observation (Spittler 2001) in laboratories, at workshops and in offices in Brazilian and German marine science institutes and universities were crucial to better understand the dynamic forces of non-humans and humans in the (de-)stabilization of MCOs. By using go-alongs, I was able to accompany researchers and technicians in their daily routines including translating data or tracking the floats in the open ocean via software. I accompanied them from the subway station to the university, to meetings with colleagues, lunch and coffee breaks, into the laboratory and workshops, while at the same time asking them questions based on my interview guideline. While doing so, some natural scientists confided their entanglements, sometimes emotions for *their* floats and lived experiences with the latter. Participant observation allowed me to dive into the working routines of researchers and technicians, such as testing sensors in the laboratory in salt and fresh water, deploying floats, or using software to track the floats in the vast ocean. To collect data on MCOs beyond Brazil and Germany, which cases were predetermined by the research project’s structure this research was embedded in, and to include the global dimension of MCOs, I participated in (inter-)national (BGC-) Argo meetings.^6^ During these meetings the European and global Argo teams discussed data, memberships, and new float deployments based on the current options to deploy them, for example, which research cruises can deploy floats in which geographical part of the ocean. Moreover, I followed negotiations between scientists and *Sea-Bird*, the latter is a float and sensor manufacturer based in the US and learnt from the difficulties and uncertainties which the scientists experienced with this manufacturer, as the company enjoys a monopoly in this area due to the nature of their highly specialized and miniatured sensors. Other floats I followed online through their GPS signal and their raw data which was transmitted via satellite and can be accessible to everyone using the *OceanOPS* platform (OceanOPS 2024). I followed nine floats during their activation and deployment on a German research vessel in the North Atlantic and Labrador Sea and witnessed difficulties in activating them which involved either removing a heavy magnet or connecting them to software, while simultaneously balancing the vessels movements and noisy environment. Finally, I followed some floats until their death, meaning the end of their battery life which lasts approximately 150 cycles, or three to five years depending on the depth to which they profile and the surface water density in which the float is operating. After this, they remain on the seabed. Only a very limited number of floats are recovered, maintained, and redeployed; and this is dependent on their location and recovery options by a vessel close-by.

In addition to participant observation and go-alongs, I also conducted 38 interviews with scientists and technicians. The interviews enabled me to learn about the complexities of geopolitics entangled in MCOs, the scientist’s and technician’s challenges with technologies, such as sensors or software, and the reasons for their personal involvement and motivation in and for marine science-making. Findings from interviews are referenced throughout the article with “Int-number” to maintain the anonymity of the interviewees.

The interviewees provided verbal informed consent to conduct the interview and publish the findings. After the data collection, field notes, photos, videos, and interviews, were transcribed and coded with *Atlas.ti*, a software for qualitative data analysis. The exploratory approach of the research allowed me to start with a set of created codes and enabled further codes to be added throughout the coding process. In line with Miles et al. (1994), I followed a thematic analysis and used a theme based coding scheme, which allows themes identified in the data to be transformed into codes both deductively and inductively, subsequently discovering patterns of themes in the data.

The identified patterns helped me analyze these themes against the backdrop of Barad’s concept of agential realism. Thinking with Barad’s (2007) agential realism, MCOs are not separated from their social or political contexts, as the following section illustrates. The findings shed light on how MCOs as intra-actions are (de-)stabilized through the dynamism of non-human and human forces.

## (De-)stabilizing marine carbon observations: Findings

The purpose of this study is to understand the actors involved in knowledge production processes of MCOs and how they manage associated challenges and uncertainties. Having conceptualized MCOs as intra-actions within the apparatus of marine knowledge production processes, I will take a further step by providing an analysis of the (de-)stabilizations of MCOs through the inseparable forces of non-humans and humans. Unfolding the dynamism of these forces is critical, as they provide new insights into the events situated within social and political contexts (de-)stabilizing urgently required MCOs for climate change scenarios.

### Stabilizing MCOs through communication, dedication, and accessibility

The core research aim was to identify the main (de-)stabilizations of MCOs. The emerging patterns through thematic analysis which stabilize MCOs are communication between the float and scientists or technicians, the dedication of scientists to translate the raw data outside their working hours, and the global accessibility of data. Only through these dynamic forces of non-humans and humans, are MCOs stabilized as the following sections will explain.

#### A human speaks to a float and the float replies: Stabilizing MCOs through two-way communication

In the past, it was not possible to communicate with the float after its deployment in the open ocean. Now through technological improvements, such as GPS, humans and floats can exchange via two-way communication. This marks a notable transformation in the apparatus of marine knowledge production. The floats are now integrated into a continuous cycle of communication and adjustment, whereas they were fully solitary sensing units. Approximately every ten days, they surface to transmit collected data and receive updated instructions via satellite, as one technician reported: “then I call them and say, “what are you doing?” So, it always depends [on the error], it's […] often things like, [the float] really didn't send data or is under the ice. […] these are often things that I can change through this two-way communication, then I can send commands to the float, and there’s a lot possible, because these are often little things like something with the hydraulics, these are limit values that you change or mission parameters” (Int-3, German male junior technician). By asking the float about its feelings, the affective and communicative entanglements of the dynamic float-human forces become visible.

Moreover, the float can send error codes to the scientist, who can then correct these. The scientist can also send commands to the float to change the depth and days of drifting, e.g., from 2000 to 4000 m depth or surfacing every five days instead of ten (Int-3, German male junior technician; Int-29, Brazilian male senior marine scientist). Scientists or technicians involved in data collection and analysis of MCOs, usually observe the deployed floats via an online tool (field notes 10.06.2022). The float’s activities are saved in the online tool every time they surface. One technician said: “I observe all the floats we have, I view them regularly, and I also have a fixed rotation for it. […] I write down when something seems strange, but I also write down when everything seems ok. So, every time I look at a float, there is a kind of continuous checklist for this float” (Int-11, German male junior technician). The technician showed me the software tool on his computer and explained that he is using a color code indicating green for “the float is fine”, orange for “task”, and red for “inoperative” (field notes 17.06.2022). He added, “[red] basically means that there is no data at all. So, then you can just look, can I do something about it or not for the time being, or is there perhaps something that I can pass on to the manufacturer or […] is it simply the final opponent, in the sense of not providing any data and that is the worst thing that can basically happen” (Int-11, German male junior technician). In the red and orange cases, the two-way communication can potentially solve errors, such as failed data transfers or wrong drifting times (Int-11, German male junior technician). Here, an agential cut of what information is included and excluded is enacted within the apparatus of marine knowledge production. The status of the float’s functioning is a material-discursive entanglement of oceanic matter, sensor, software, and technician.

The communication between floats and humans mediated by GPS and satellite stabilizes MCOs. Through the two-way communication, scientists and technicians can reach the floats although they are out of human reach and sight, since the float solitarily drifts through the ocean measuring biogeochemical elements. In return, the floats can communicate with the scientists or technicians by sending them error-codes. Yet, much of the float’s operation remains outside of the human eye. These unseen episodes are an agential cut in which neither the natural scientists and technicians, nor I as ethnographer can observe the whole material-discursive apparatus. Thus, the two-way communication does not represent simple interaction between technicians or scientists and the floats but mirrors the dynamic intra-active becoming of MCOs from a Baradian perspective. Only through the dynamic forces of oceanic matter, floats, sensors, communication, software, and humans, MCOs are enacted and stabilized. Yet, this stabilization does not signify the elimination of failure, but rather a provisional configuration of entanglements within the murky waters of ocean sensing.

#### Volunteering and dedication for stabilized MCOs

During a float deployment in the North Atlantic which I was part of, I observed a moment of silence, of sadness, of togetherness, when everyone who participated in the deployment, both scientists and crew members, witnessed the float disappearing into the vast ocean. These moments allowed me to discover how humans are connected to the technologies they work with on a daily basis (field notes 19.08.2021) and how these entanglements are affective and relational. My observations at sea spurred me to also discuss the entanglements my interlocutors had with their technology on land. A scientist who I interviewed in his office mentioned that he named his floats before deployment (Int-18, German male senior marine scientist). Another scientist always marked the float before deployment with a sticker of the respective cruise to leave a physical trace of where and in which context the float has been deployed (Int-37, Brazilian male junior marine scientist). The floats all have a series number and can be tracked via an online tool and GPS. Another scientist told me that she “stalks” the floats online to see “what they are up to” (Int-1, German female senior marine scientist). These observations of care e.g., naming and stalking the floats, enact a constant co-becoming of new phenomena as the scientists are trying to be part of MCOs through these material-discursive entanglements.

Thus, scientists are constantly in touch with their floats. They also invest a significant amount of (over)time and effort in procuring funding for the floats, related technologies, data storage, translation, and analysis (field notes 14.06.2022). Due to the lack of funding for MCOs as a whole, but especially for staff for data translation, the marine science community has mobilized themselves into working groups to enable national, European, and even global MCOs by working voluntarily on data translation (Int-1, German female senior marine scientist; Int-3, German male junior technician; Int-4 and Int-5, both German male senior marine scientists, and Int-18, all German male senior marine scientists; Int-25, Brazilian female senior marine scientist). Since the float sends its data in the form of electronic signals via satellite, only raw data arrives at the data centers and this needs decoding into physical units (Int-1, German female senior marine scientist), as one scientist explained: “[…] we have very confusing data formats that can’t be read at all. These are binary formats, you first need software to decode them and then you get physical units that you can read, but of course that's not the whole story, depending on what kind of sensor you have, you have to make different corrections” (Int-2, German female junior marine scientist). These corrections are already a minor refinement, as they involve a second review ensuring data quality and adequate metadata, which facilitates further use of the dataset (Int-4, German male senior marine scientist). Through the scientist’s voluntary work and dedication to transform the data into usable knowledge, MCOs are enacted. Only through dynamic processes of non-human and human forces high-quality data is made available to the public (Int-3, German male junior technician), which mirrors Barad’s becoming of reality as a dynamic process. Without scientist’s work, the float’s data would be “useless” (Int-2, German female junior marine scientist). Scientists are not being paid to translate the raw data. Instead, the Surface Ocean CO2 Atlas (SOCAT) brings together a group of scientists who voluntarily translate and check the submitted raw data of MCOs. One scientist explained that “the data records are increasing. There is also more and more data that is poorly measured and gets suspended […], that’s a kick in the ass for every volunteer, because they all do it in their free time on the side” (Int-4, German male senior marine scientist), emphasizing the scientists’ firm conviction to their work on MCOs. What these interview quotes moreover show are the Baradian diffraction enacted across disciplinary boundaries. In the joint effort of the scientists, the knowledge production processes become only visible and together they achieve the outcome of making the data understandable and usable.

Besides the decoding of data, scientists write their own software, often in *MATLAB*, which is a programming platform (Int-1, German female senior marine scientist; Int-19, German male senior technician). They voluntarily program these software applications and make them freely available to anyone interested in using it. These applications are particularly necessary if marine forces, such as boundary currents, interfere. “[If the floats] are traveling in the Gulf Stream, where the velocity is very high, then the movement of the floats, which drift with the current, is much more violent and they don’t stay as long in areas where you actually want to collect data. If you want to have just as much data in boundary currents as in the interior of the ocean, we are now writing software […], in which you can virtually predict these” (Int-1, German female senior marine scientist). By providing this voluntary work, such as decoding data, programing software, and making these publicly available, MCOs can be stabilized to a better extent. But more importantly, by developing the software, the scientists want to avoid an agential cut, meaning the software serves as an intra-acting force aiming to not lose track of the float and thus the data. The careful approach of the scientists, which targets the float’s continous operation, highlights the material-discursive and dynamic becoming of MCOs.

#### Stabilization through globally available and accessible MCOs

So far, little is known about nation states’ practices and reasoning of observing marine carbon and of providing the required financial, technological, and human resources to do so. Thus, the sole focus on humans and floats overlooks the important processes of geopolitics as part of MCOs, and as both non-human and human intra-acting forces, which are inseparable. These include the availability and accessibility of MCOs, entanglements with the EEZ or a nation state’s financial contribution to the Argo program. To join the European Argo program, a nation state is required to have a long-term engagement with Argo at the country level, and should have developed a national Argo program with the deployment of a minimum of three floats each year, and contribute €30,000 for the central coordination management, often paid at the Ministry level (field notes 08.04.2021). This potentially leads to the exclusion of some countries due to the high monetary investment required (see 4.2.3).

Besides the accessibility to the Argo program, the available contributions of MCOs to the Global Carbon Budget and the IPCC were frequently emphasized in the interviews conducted and seen as a crucial basis for climate change negotiations (Int-4; Int-5; Int-12 and Int-18, all German male senior marine scientists; Int-14, Colombian female junior marine scientist). These global climate governance mechanisms are not simply an outlet for climate data or a pre-existing reality, but they are part of the mutual constitution of MCOs. MCOs are a material-discursive mediator between global climate governance, climate forecasting, carbon budgeting, and marine environments. All data from Argo floats is available online and “[…] anyone could actually use them” (Int-1, German female senior marine scientists). As such, MCOs and the public access to its virtual data can constitute mediation and sharing of knowledge on climate change and the ocean’s health. In practice, any person with access to the internet can use the data on MCOs. The global availability and accessibility of data enacts the material-discursive field of climate governance and co-constitutes the open-ended process of knowledge production on the ocean and climate change. Scientists, technicians, floats, sensors, software, data, the translation of data, and climate governance mechanisms are all part of the apparatus constantly enacting MCOs.

Although the floats for MCOs are geographically unevenly distributed among nation states and the ocean (see Funding, (national) boundaries and inequalities as destabilization of MCOs), the open access, availability, accessibility, and sharing of data and knowledge constitutes an enacted agential cut in form of an inclusion. The translation and standardization of data supports and mediates epistemic equity as even countries who are not part of the Argo program can use the data. The active role of MCOs in its mode of knowledge mediation, for example climate change scenarios and respective global policies can be described as, “matter comes to matter” in a Baradian sense (1998) and stabilizes MCOs.

### Destabilizing MCOs through climate change, lost knowledge, and (national) boundaries

Having identified the main forces stabilizing MCOs, the subsequent sections examine the destabilizing forces of the latter. Climate change forces, such as strong currents or marine life, lost knowledge caused by the retirement of scientists or technicians, and inequalities in funding, science systems, and along national boundaries are inseparable forces destabilizing MCOs.

#### An antithesis: The forces of climate change destabilize MCOs for climate change scenarios

Paradoxically, climate change forces themselves can lead to a destabilization of MCOs. One scientist explained that climate change has caused mild winters, which has led to different diving depths of the floats, ergo to a variation from 500 to 4000 m water depth leading to distorted observations (Int-1, German female senior marine scientist). Moreover, ocean currents and eddies, which are fast-swirling water columns, can sometimes lead to a permanent loss of the float’s GPS signal for a certain amount of time and consequently to an agential cut (Int-7, German male senior marine scientist), because the float can no longer send its data and humans can no longer identify the location of the float leading to periodical loss of data and communication. Again, an agential cut in the form of an exclusion emerges, in which the boundaries between observer and observed become visible. Neither humans nor non-humans within the apparatus can reach each other, highlighting the constant uncertainty and destabilizations in knowledge production processes of MCOs.

Sometimes, floats also diffract (inter-)national boundaries as they drift into the Exclusive Economic Zones (EEZ) of countries, which usually require prior authorization of MCOs through the Argo program (Int-1, German female senior scientist). Although these drifts are caused by uncontrollable ocean currents, the responsible scientists in Germany must inform the countries which prohibit foreign floats in their EEZ, such as Brazil and Russia (field notes 09.06.2022). Similar observations have been reported for the South China Sea where multiple countries have competing territorial interest, and the presence of foreign floats could be viewed as a potential challenge to sovereignty, leading to restrictions (field notes 17.08.2022; see also Funding, (national) boundaries and inequalities as destabilization of MCOs) and a destabilization of MCOs through these agential cuts of prohibiting or confiscating floats.

The floats themselves and especially their sensors are likewise affected by marine life, such as mollusks, which can colonize the surface and the sensors of the floats, especially in the upper layers of the ocean, where light permeates. This can lead to gradual inaccuracies in the salinity sensors of Argo floats, requiring corrections with data obtained from SOOP measurements and laboratory-prepared standards (Int-11, German male junior technician; cf. Lehman 2018; Roemmich et al. 2001), resulting in destabilized MCOs. Similarly, some interviewees mentioned “flawed data” (Int-1, German female senior scientist; Int-4, German male senior marine scientist) mostly caused by climate change effects as a challenge for MCOs. These are caused by sensor malfunctions and technical issues and can lead to periodic or constant agential cuts within MCOs, because the data is unreliable. Scientists then need to review the data closely and decide if it can be published or needs to be erased. “And if you do that, then you want to do it fairly, that means you have to look at this data set, you don’t just say no and say “suspended, we won’t take it”, but you have to at least write something about why [the data is] suspended” (Int-4, German male senior marine scientist; cf. Int-20, Brazilian female senior marine scientist), which increases the scientist’s workload drastically. These agential cuts diffract the knowledge production process of MCOs as unexpected outcomes, such as overtime or unusable data are enacted, highlighting the dynamic becoming of reality. Thus, the ocean’s geographic features itself, climate change forces and resulting “flawed data” and their material entanglements can destabilize MCOs.

MCOs are constantly (de-)stabilized through diverse forces at play in open-ended processes. In this case these forces are both human and non-human and predominately caused by climate change and physical characteristics of the sea pointing to the ocean’s relational ontology of responding, resisting and fluctuating. Looking at these dynamics through a Baradian lens makes sense in better understanding the complexity of MCOs and to take agencies beyond the human seriously. MCOs are constantly becoming, they are not fixed truth as they are in constant intra-action with marine life, humans, and technologies.

#### Lost knowledge and an engineered death as destabilization

As a result of limited permanent funding in academia, the attrition rate of staff in MCO research teams is high. With these changes in personnel, much of the crucial knowledge to fund, implement, and analyze MCOs disappears. One oceanographer reported: “There will simply be fewer and fewer people who can evaluate this. Of course, there will certainly be autonomous systems that will be able to evaluate this themselves at some point in the future. But as long as that doesn’t happen, there will be problems at some point. Data is always collected, but there is no one who can evaluate it because there are no jobs. Or there are no jobs created, and I think that's the problem, that you’re really tied to these funds […].” (Int-8, German female senior marine scientist), highlighting the loss of knowledge and limited funding in MCOs. This diffraction is not only a loss or rupture, but it is rendered as productive in a Baradian sense, as it leaves space to think the old anew. The absence of this knowledge continues to intra-act with what remains. In this case it uses the diffraction to question the science system and enacting epistemic consequences and constant becomings. The scientist continued: “And that’s what I think should change in the future, that this scientific system should be reconsidered at some point, that we really need to create permanent positions for people who can continue to do this and not always just through third-party funding” (Int-8, German female senior marine scientist), referring not only to the intersection of the German academic system and the knowledge of analyzing data, but also to the constant (un)becoming of scientists and technicians in the apparatus of marine knowledge production.

Retirement constitutes another agential cut through which essential knowledge disappears. One technician at a German research institute put it like this: “When I leave, this place will probably be closed. There will be no successor. Retrenchments are being made everywhere” (Int-17, German male senior technician). A researcher added: “Then they just say, now the data is there, and nobody takes care of it and at some point, nobody knows how to handle this data and how to process it anymore” (Int-8, German female senior marine scientist). Thus, the complex and often long-standing knowledge production processes and knowledge transfer of MCOs will be lost. At the same time the diffraction of lost knowledge and epistemic discontinuities can cause misalignments in ongoing MCOs, e.g. incorrect float maintenance or deployments. The exclusion of this knowledge and practices also represents agential cuts, which are ethically charged in Barad’s ethico-onto-epistemology as the material-discursive shift leads to reconfigurations of what reality is becoming.

Besides frequent job changes, limited funding, and retirement as destabilizing factors for MCOs, there is also a lack of technicians to stabilize MCOs through the calibration of sensors, and maintenance and deployment of floats, as one junior marine scientists explained: “We actually have a shortage of technicians and you notice that, sometimes there are a lot of tasks that could easily be delegated to technicians […], but which you then have to do yourself […] and that of course costs a lot of time, which you then can’t spend on your actual research” (Int-11, German male junior marine scientist). The lack of technicians to support the scientists in their research was reported in all four marine science institutes that the research was conducted in, both in Germany and Brazil, pointing to a common phenomenon and the inseparability of intra-acting forces within the apparatus that is constantly reconfigured.

Another destabilization of MCOs constitutes the actual death of a float, meaning their human-engineered lithium battery, after which, they remain on the seabed (Int-18, German male senior marine scientist). Argo floats are made from plastics, lead, aluminum, copper, zinc, metals, and lithium battery. After the depletion of the battery, a float sinks to the seabed, introducing pollutants to marine life (Riser and Wijffels 2020). During fieldwork, I was puzzled by how such a human engineered float could simply remain on the seabed in times of climate change, ocean pollution, and limited resources. The large number of floats on the seabed raises concerns in the scientific and non-scientific community about their environmental impact, as some interviewees reported (Int-3, German male junior technician; Int-4, German male senior marine scientist). Isn’t there another solution to avoid the floats becoming a sinking relic? Yet, Argo argues that “presently there is no method of observing the subsurface global ocean that is less environmentally damaging and more cost effective than Argo” (Euro Argo 2024). A study by Riser and Wijffels (2020, 9) found that “the chemical species injected into the abyssal waters during this process represent generally infinitesimal amounts in comparison to the natural and anthropogenic fluxes of these substances”. Thus, despite the environmental impact and the missing human engineered solution, Argo floats are currently the most effective instrument for urgently needed MCOs. However, the death of a float always contributes to the destabilization of MCOs, emerging out of the material-discursive forces of non-humans and humans. Simultaneously, the application of Barad’s agential realism to MCOs underscores the inseparability of non-human and human forces. Especially MCOs as scientific practice aiming for climate change mitigation cannot be separated from the environmental and socio-economic entanglements and its ethical implication highlighting Barad’s ethico-onto-epistemology.

#### Funding, (national) boundaries and inequalities as destabilization of MCOs

**Fig. 3:**
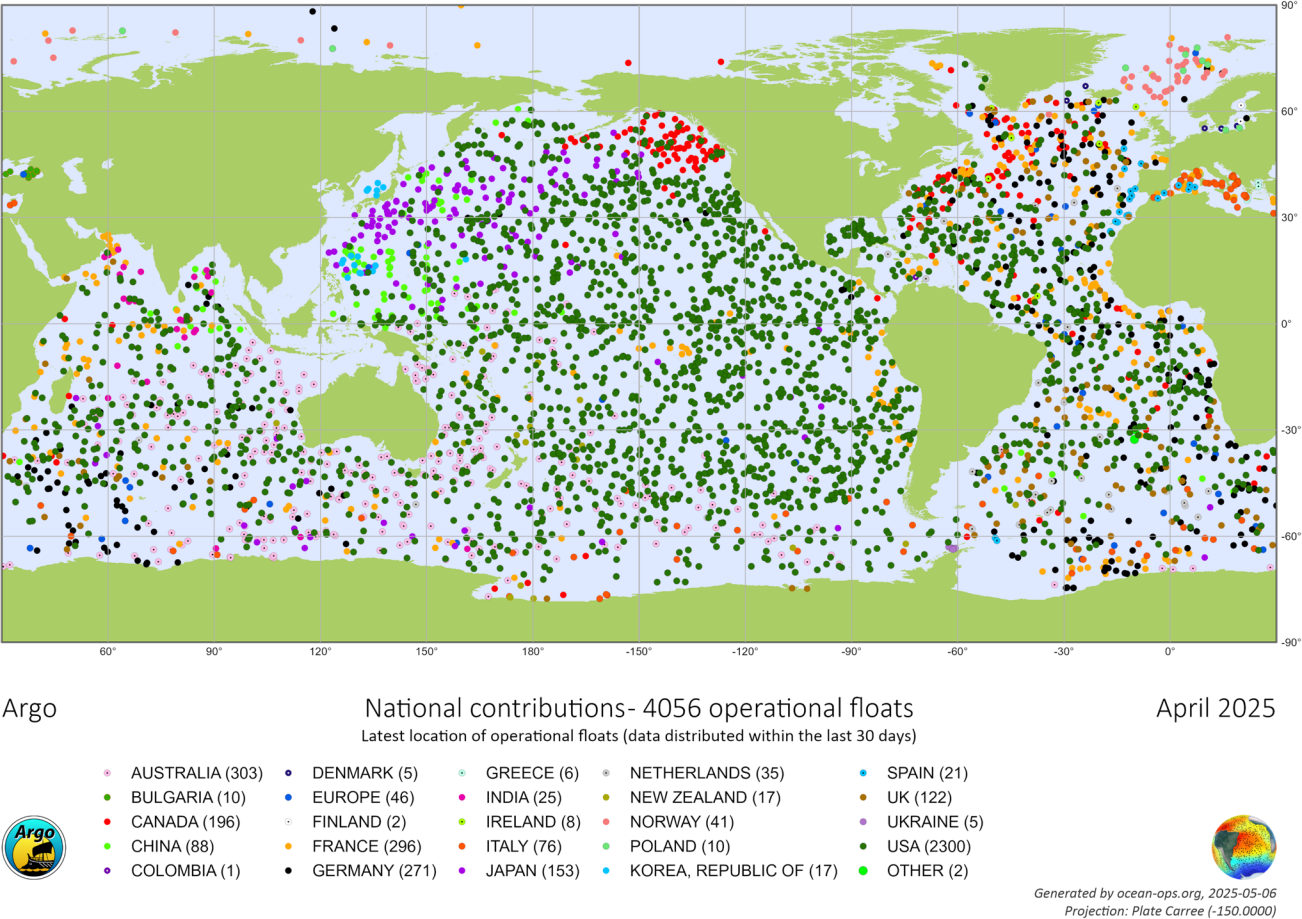
National contributions to the global international Argo monitoring network with about 4,000 active Argo floats (Argo 2025)

MCOs are situated in a contested vacuum of epistemic inequalities (cf. Dotson 2014; Fricker 2007), data accessibility, demand for modelling, climate change scenarios and a complex bond between non- human and human forces. The Argo program consists of diverse national contributions of floats aiming to provide data to understand the ocean’s role in the earth’s climate. Yet, looking at the global distribution and contribution of different nations to the Argo program (Fig. 3), countries in Africa, Latin America, or Southeast Asia, are highly underrepresented and if represented, only with a very limited number of floats, e.g., Peru currently has only one active float compared to the US which operates more than 2000 floats. The map also displays a very limited number of floats in coastal areas, pointing to a prohibition or restricted permission of deployments in EEZs. Questions emerge on the geopolitical interests of nation states (cf. Melvin et al. 2023), (epistemic) inequalities (cf. Fricker 2007) of MCOs and knowledge on climate change.

As figure three shows, many nations are not part of the Argo program and thus, not directly part of the apparatus of marine knowledge production. The inclusion and exclusion of nations and respective scientists point to inequalities in the international science system and can be explained by the lack of funding of MCOs (Int-10, Brazilian male junior marine scientists; Int-12, German male senior marine scientist; Int-14, Colombian female junior marine scientist; Int-20, Brazilian female senior marine scientist). Interviewees mentioned a lack of funding, staff, and time as limitations for observing marine carbon (Int-1, German female senior marine scientist; Int-3, German male junior technician; Int-4; Int-5 and Int-18, all German male senior marine scientists). Funding as part of the apparatus enacts MCOs. Thus, limitations in funding for staff and material are agential cuts that decide which nations, regions, and parts of the ocean matter in the constant (co-)becoming of MCO and global knowledge on the ocean and climate change. In Germany, the Federal Ministry for Digital and Transport funds the atmospheric carbon measurements, which the state also needs for its national carbon budget calculations. There are fixed stations on land where measurements are taken, but there is no continuous funding for oceanographic measurements to date (Int-1, German female senior marine scientist; Int-4 and Int-5, both German male senior marine scientists; Int-6, German male senior technician). Float procurement, for example, is therefore always subject to third party funding and needs constant renegotiation. Such obstacles impede long-term and sustainable MCOs (Int-1, German female senior marine scientist; Int-5, German male senior marine scientist). “You also must have contacts to the budget department, the procurement department, so that you can comply with the relevant rules. Even if I know I have money next year, I can only order next year and I have to make sure that I spent all the money by the end of the year, because otherwise the money has to be returned to the Ministry of Finance” (Int-1, German female senior marine scientist), explained one oceanographer.

Moreover, (national) boundaries and EEZs can produce constant or periodical agential cuts of MCOs enacted through geopolitics. National borders and EEZs play a central role in enacting MCOs. Some countries, such as Russia have asked for bilateral communications if an Argo float enters their EEZ. In such a case the national program focal point (BSH for Germany) acts on an automated alert system installed at *OceanOPS* and contacts the national Argo focal point in the respective country and informs them accordingly (Int-1, German female senior marine scientist). This boundary-drawing practice in territorial terms translates into an epistemological one as it defines who observes, defines, and shares knowledge on MCOs. MCOs in the Arctic are of crucial importance for climate change scenarios. Yet, so far, only limited observations are possible, because the Argo community does not “know whether we will be allowed to do anything in the Arctic in the next few years. Because the European part is almost all territory of Russia. […] we cannot deploy floats directly in a foreign EEZ without authorization but have to rely on the float drifting into the right area. In the Arctic, where we would like to have measurements from the Russian shelves, we can currently only deploy just off the Russian border in Norwegian waters and hope that the currents will do the rest.” (Int-1, German female senior marine scientist). Some countries prevent globally required MCOs for geopolitical reasons, such as security concerns, thereby reconfiguring the apparatus of marine knowledge production intentionally.

The current distribution of floats and data centers also points to a financial and geopolitical inequality globally. France manages the data center for all European Argo data, while the US has its own center (Int-1, German female senior marine scientist). This distribution points to a hegemony of the US, as one scientist reported: “Argo was an American project, it’s dominated by American floats and the American community also dominates the technology, and the companies around it. The Americans would never buy from others, they have a much different attitude to us, they wouldn’t buy French or Japanese floats, it’s out of the question, they’re certainly not allowed to with NSF [National Science Foundation] and NOAA [National Oceanic and Atmospheric Administration] funding” (Int-5, German male senior marine scientist). Similarly Lehman (2018, 74) found that the US is the largest funder of Argo. In particular, the navy is interested in areas of the sea “where it has marine interests, not just off the US coast but also […] in the South China Sea”. The interest of one country and the non-participation in the Argo program by other countries as an agential cut highlight that MCOs are not neutral but enacted by historically uneven power dynamics. Due to the highly specialized nature of manufacturing sensors for floats, the US-owned company Sea-Bird has the monopoly. In recent years, however, the manufacturer prioritized orders from North America, delaying dispatches to other countries (Int-5, German senior marine scientist). Hence, this domination of the US in MCOs can potentially lead to their destabilization in (and from) other nations, which do not have the respective knowledge, funding nor human resources. Barad’s ethico-onto-epistemology furthermore makes these inequalities visible. Science is not detached from the material practices and the power asymmetries of global MCOs – a site of contested and constant (un-)becoming of reality. Both, the paradox of contributing to climate change and marine pollution while simultaneously trying to solve these with MCOs (see Lost knowledge and an engineered death as destabilization) and the financial, political, and geographical dominance of certain nations in MCOs can be analyzed as technological fixes (Harvey 2003) and may reinforce geopolitical, capitalist hierarchies, and ecological risks rather than solve these.

As the previous sections have shown, a dynamism of inseparable intra-acting forces is part of destabilizing MCOs (Table 2). The final section will summarize these findings and provide an outlook for further research needed.

**Table 2:** (De-)stabilizations of marine carbon observations (own elaboration based on Barad (2007) and own data collection (Int-1 – Int-38; field notes)).

Stabilization	Destabilization
Volunteering of scientists in making data understandable and accessible	Climate change effects and flawed data
Two-way communication between float and human	Lost knowledge and death of floats
Mediation role of MCOs on climate change and policies	Funding of MCOs
Global data accessibility free of charge	National borders including EEZs

## Dynamic (de-)stabilizations of MCOs: Concluding remarks

The article took qualitative data from fieldwork on a research vessel and in Brazilian and German marine science institutes which produce knowledge on marine carbon as a vantage point for this article, to ask these questions: How do scientists responsible for determining marine carbon navigate the methodological and technological challenges and uncertainties they encounter? How do they collect, interpret, and share data, or, in other words, how is knowledge about marine carbon (socially) produced? And how are the forces of non-humans and humans implicated in (de-)stabilizing MCOs? In short, how are MCOs (de-)stabilized?

By following floats, extending observations from land to sea, and incorporating usually inaccessible locations such as the deep sea and satellites, the research was able to provide valuable insights into the complexity of MCOs, highlighting the co-constituting nature of non-human and human forces in shaping our understanding of oceanic carbon processes and its entangled material-discursive practices. I argue that Karen Barad’s (2007) concept of agential realism provides a useful framework for understanding MCOs. Rather than viewing different components or actors involved in MCOs as separate entities interacting, intra-action emphasizes their mutual co-constitution.

In my understanding of Barad’s apparatus as a semi-permeable membrane, the non-human and human forces at play cannot be separated. The dynamism of inseparable forces is ever-present. Apart from shedding light on (de-)stabilizations of MCOs, the analysis also identifies diverse (un-)becomings, doings, and diffractions within the apparatus of marine knowledge production processes. The becoming of (marine) scientists and technicians, but also their doings by co-enacting knowledge on climate change, as well as their unbecoming through retirement or budget cuts is apparent when thinking with Barad’s concept of agential realism.

The study furthermore examines the material-discursive, mainly geopolitical forces (de-)stabilizing MCOs, which have previously rarely been studied. This highlights how political and geographical forces are essential in enacting MCOs, as they are co-constituting scientific practices and technological systems. Yet, the research highlighted global inequalities and raised questions about decision-making, access to funding, and exclusion. The (de-)stabilization of MCOs appears to be shaped by power relations, funding structures, and governance mechanisms. These dynamics may also obscure environmental or political harms. Science-making in MCOs is more than just data collection as it is always entangled with materiality, emotions, ethics, and responsibility. Producing knowledge on MCOs is a material-discursive practice in which MCOs serve as mediator of what the ocean is and becomes in the context of global climate science-making and governance. Moreover, Barad’s lens of agential realism made the usually unseen labor of scientists and technicians, the hidden hierarchies of national, financial, and oceanic boundaries, and the loss of diverse types of knowledge visible.

Moreover, my doings as a researcher show the material practice of knowledge production and its ethical and political implications, adding to Barad’s ethico-onto-epistem-ology. With a diffracted understanding of me as a social scientist conducting research on natural scientists’ knowledge production, I am contesting positivist understandings of subjectivity and objectivity, but I also enact agential cuts within the apparatus of knowledge production by observing and including certain phenomena, while others remain excluded.

I invite you once more to imagine 4000 Argo floats moving up and down the ocean. Perhaps you thought, much like I did, that measuring marine carbon would be straightforward before reading this. But measuring marine carbon is by no means straightforward; they, to various degrees, take place in murky waters. The paper identified challenges and often-times overlooked dynamics within MCOs, clarified some of these and highlighted the intricate co-constitutions among various forces entangled in MCOs, yet it may have inadvertently revealed even more challenges than anticipated. Especially the material afterlife of floats and the environmental, labor, and ethical costs of MCOs, requires further research. Ultimately, navigating these murky waters by both floats and scientists underscores the ongoing need for a deeper understanding of how marine carbon research does (not) work.

## Data Availability

Due to the nature of this research and for ethical reasons, detailed interview transcripts will not be shared publicly, so supporting data are not available. The author guarantees the pseudonymization of interviewees.

## Footnotes

1 Most of these floats are standard Argo floats measuring temperature and salinity. Currently, only 397 BGC (biogeochemical)-Argo floats with pH sensors are active. The low number of BGC-Argo floats can be explained by their expensive acquisition (OceanOPS 2024). Depending on the equipped sensors of the float, the costs for a float range approximately between $20,000 and $150,000. A standard float with temperature and salinity sensors is much cheaper than a sensor with more advanced tools for measurements, such as pH, nitrate or oxygen sensors.

2 (BGC-) Argo floats are not the only instruments to measure marine carbon, biogeochemical elements and hydrographic properties. Other instruments include drifters, gliders (Byrne et al. 2010), or autonomous marine carbon observation systems on so-called Ship of Opportunity Lines (SOOPs). SOOPs are merchant vessels that voluntarily carry the observation system, allowing marine scientists to collect data every time the SOOP returns to its port of registry. These carbon observation systems measure the ocean surface’s salinity, pCO2, temperature, alkalinity and pH value (Steinhoff et al. 2019; Int (Interviewee)-4, Int-5, German male senior marine scientists; Int-17, German male senior technician). Due to the limited number of SOOP lines globally and respective interlocutors for these types of marine carbon measurements, this study focuses on marine carbon observations via (BGC-) Argo floats.

3 Fieldwork was conducted by the author in an interdisciplinary research project funded by a German ministry and carried out jointly by Brazilian and German (marine) science institutes.

4 The interviewee’s geographical location and predominant nationalities of interviewees being German and Brazilian can be explained by the research project’s structure the research is rooted in (see funding). The case selection was predetermined and could not be changed.

5 Usually, German marine science institutes, such as the Helmholtz Centre for Ocean Research Kiel (GEOMAR), the Federal Maritime and Hydrographic Agency (BSH) in Hamburg, and the Leibniz Institute for Baltic Sea Research Warnemünde (IOW) negotiate with funding agencies, mainly ministries, such as the German Federal Ministry for Digital and Transport, for the procurement of floats.

6 These meetings included the EU Argo Workshop in April 2021, the Global BGC-Argo Fleet Workshops from May to June 2021, DArgo2025 Meeting in May 2021, G7 Resource Strategy Group Workshop in March 2022, Euro-Argo Management Board Meeting and the Argo Germany User Meeting in June 2022, ICOS (Integrated Carbon Observation System) Germany Annual Meeting in December 2022.
